# Epidermolysis Bullosa Simplex With Dystonin Gene Mutation: First Reported Case in Saudi Arabia

**DOI:** 10.7759/cureus.43206

**Published:** 2023-08-09

**Authors:** Mohammed Al Towijry, Abeer Mohammed M Alanazi, Fatma Eldesoky, Yousef H Alharthi, Ibrahim Abdullah S Albalawi

**Affiliations:** 1 Department of Pediatrics, King Salman Armed Forces Hospital, Tabuk, SAU; 2 Department of Dermatology, King Salman Armed Forces Hospital, Tabuk, SAU

**Keywords:** child, homozygous, gene mutation, skin disease, epidermolysis bullosa simplex

## Abstract

We report a case of a 3-year-old Saudi female patient as the first case reported in Saudi Arabia who is homozygous for dystonin c.3370C>T, p.(Gln1124*). At the age of 3 months, the girl started to develop numerous vesicles and bullae involving the dorsum of the feet that were not on a pressure site, with remission and aggravation, but she had no mucosal lesions or nail affection. The patient was diagnosed with epidermolysis bullosa simplex.

## Introduction

The term epidermolysis bullosa (EB), also known as "butterfly child disease," "cotton wool baby syndrome," and "crystal skin syndrome" refers to a spectrum of hereditary blistering conditions that present as skin and mucous membrane blisters following mild injury or trauma [1]. In the United States, the overall incidence and prevalence of the disease are approximately 19 per one million live births and 8 per one million population, respectively, with no preference given to gender or race, classifying it as a rare or orphan illness [2].

Due to the fact that there are now known mutations in 18 distinct genes that cause defective structural and functional cellular adhesion, the phenotypic spectrum of EB is highly broad. Some variants of EB also cause blistering of the skin and mucous membranes, as well as the involvement of the gastrointestinal (GI), respiratory, and genitourinary systems, as well as very aggressive squamous cell carcinoma, skin adnexa, hair, enamel, and nail dystrophy. The most common type of EB is epidermolysis bullosa simplex (EBS), which is often caused by mutations in the genes KRT5 or KRT14, encoding keratins 5 and 14, respectively. EBS usually results in blisters forming in the basal layer of the epidermis [1].

EB currently has no effective therapeutic options. beyond strict wound management and chronic pain control. The treatment of EB has evolved over time and recent advancements have introduced promising potential therapies. These include gene therapy, which targets the underlying genetic mutations that cause EB. In 2020, researchers reported the successful use of gene therapy in a child with junctional EB, using skin grafts grown from the patient's own genetically corrected cells. Further studies are needed to determine the long-term efficacy and safety of this approach. Another therapy being studied is cell-based therapy, which involves the use of stem cells to promote healing and reduce inflammation. The cells can be delivered through intravenous infusions or topically applied as a part of a skin graft. Both mesenchymal stem cells (MSCs) from bone marrow or adipose tissue and hematopoietic stem cells (HSCs) from bone marrow or umbilical cord blood are under investigation. Protein replacement therapy is also under consideration, which involves providing patients with a synthetic version of the protein their bodies cannot produce due to genetic defects, like type VII collagen in dystrophic EB or laminin-332 in junctional EB. Topical treatments are under investigation, including those that can promote wound healing, reduce inflammation, or deliver gene therapy or protein replacement therapy directly to the affected skin. Furthermore, RNA-based therapies are designed to interfere with the process of translation from RNA to protein in cells, helping to correct or mitigate the effects of genetic mutations. Lastly, small molecule therapies are being researched, which involve the use of drugs that can either promote wound healing, reduce inflammation, or potentially modulate the genetic abnormalities seen in EB. It is important to note that these treatments are currently at various stages of research and development, from preclinical testing to clinical trials. While they have the potential to greatly improve the lives of people with EB, they also carry risks and uncertainties that need to be carefully evaluated [3,4].

Out of the 18 previously known genes in EB, one may discover those coding for anchoring fibrils in skin and mucosa, adhesion cytolinker proteins, desmosomes, hemidesmosomal plaque proteins, and enzymes of epidermal development. Based on the severity of the skin blistering, there are four main subtypes of EB [1]. The primary subtypes are further separated into more than 30 distinct forms, and severity typically varies from moderate to fatal [5].

The last updated recommendations on EB diagnosis and classification were published in 2014. Recently, clinical and genetic aspects, genotype-phenotype correlations, and disease-modifying factors have all been reviewed, and concept of genetic disorders with skin fragility, of which the classical EB represents the prototype has been considered. EBS with dystonin (DST) gene mutations is a unique variant of EBS that often presents a combined phenotype of skin blistering with additional neurological symptoms due to the essential role of dystonin in maintaining the integrity of the skin and nerve cells. Mutations in the DST gene are more often associated with the more severe forms of EBS, such as EBS with muscular dystrophy and EBS with pyloric atresia. An interesting and unique aspect of DST gene mutations is that, unlike other forms of EBS, the disorder may also have a neurodegenerative component [6]. Previously reported cases with DST mutations that presented EBS manifestations are tabulated in the Appendices.

## Case presentation

**Figure 1:**
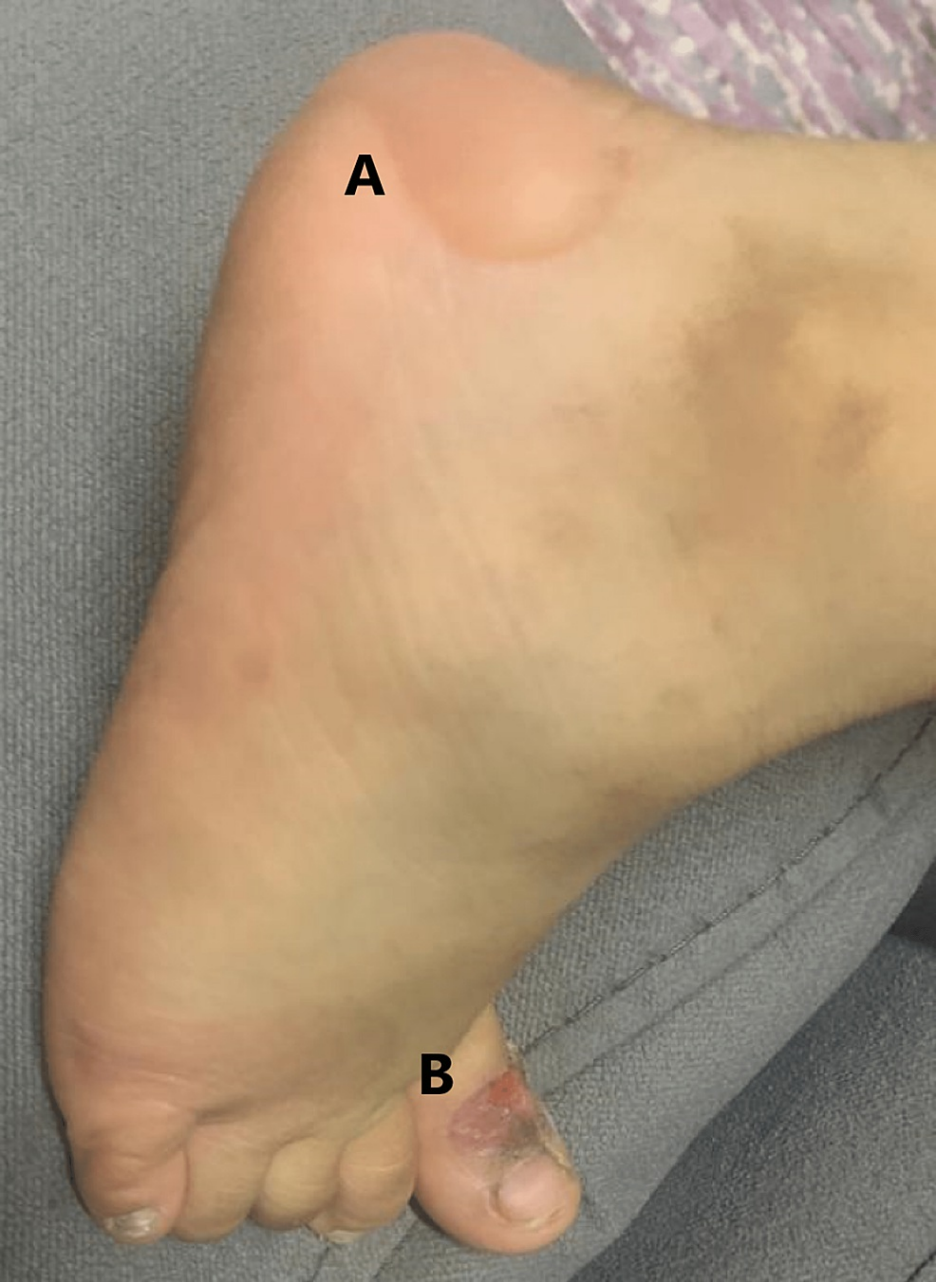
Foot of the girl showing a large bulla filled with watery fluid at the heel (A) and scars at the big toe (B).

We present a case of a 3-year-old Saudi female patient who, for the first time in August 2021, attended the outpatient dermatology clinic at King Salman Armed Forces Hospital in the Northwestern Region with recurrent vesicles and bullae on her hands and feet. Her parents reported that the initial presentation of the lesions was characterized by small, pinpoint blisters that gradually increased in size over the course of a few days. As they grew, they filled with clear, watery fluid and formed non-painful vesicles and bullae. These blisters typically formed on areas of the skin that were subjected to mild trauma or friction, primarily on the patient's hands and feet. In some instances, larger blisters would rupture, either spontaneously or due to physical contact, leading to the formation of open sores. As the healing process initiated, the blisters gradually deflated and dried out, forming a crust. Eventually, the crust would peel off, revealing a new layer of skin underneath. The newly formed skin, initially pinkish in color, would then darken over time, leading to a hyperpigmented spot. Importantly, the healing process did not lead to any noticeable scarring (Figure 1). No mucosal lesions or nail affection has been reported.

The patient had an uncomplicated vaginal delivery, was up to date with immunization, and has 4 siblings aged 6, 8, 12, and 15 years who were unaffected by the disease.

Teeth abnormality and caries started with skin problems but did not cause difficulty when eating. The patient’s parents first attempted to clean her teeth when she was between one and a half and two years of age, which proved to be extremely difficult at first. Only when she was approximately 3 years old it became considerably easier to brush her teeth. The bullae did not affect the mucous membranes in the mouth.

The patient was breastfed for a short time and was then bottle-fed until she was 24 months of age. Overall, the patient’s food intake was normal. At present, she enjoys eating soups and drinks water, milk, and fruit juices. On examination, the patient was found to have numerous vesicles and bullae filled with watery fluid involving the dorsum of the feet that were not on the pressure site starting at the age of 3 months with remission and exacerbation, no mucosal lesions, no nail affection. A genetic study for a skin biopsy was done on August 22, 2021, and revealed that the patient is homozygous for DST c.3370C>T, p.(Gln1124*).

In our case, the management was largely supportive, involving prevention of blistering, wound care, and monitoring for any secondary infections. The patient was given symptomatic treatment with potassium permanganate topical antibiotic cream twice daily until the wound completely healed. Special attention was given to the child's nutrition to ensure adequate growth and development. In addition, the child and her parents were guided on how to avoid skin trauma to prevent the formation of new blisters. The mother ensures regular follow-ups at the dermatology clinic every 3 months, adhering to her child's treatment plan. In response to the provided care, the patient showed improvement in terms of the reduced frequency and severity of blisters, and her overall quality of life was also enhanced. The child still developed new blisters occasionally. The parents were counseled about the chronic nature of the disease and the possibility of disease flares even with ongoing treatment. The parents were provided with extensive education regarding home care for blisters and were also counseled about the genetic implications of the disease.

## Discussion

EBS is characterized by the production of intraepidermal blisters. It is divided into basal and suprabasal subtypes by the extent of blistering in the epidermis. Surface blistering that appears above the epidermis's basal cell layer is known as suprabasal EBS [7]. This category comprises severe autosomal recessive variations that are highly uncommon and caused by mutations in the genes encoding the desmosomal proteins plakophilin-1 (PKP1), desmoplakin, and plakoglobin (JUP) [8].

Following an extensive literature review, this is the first Saudi case reported with DST mutation and EBS presentation. Blistering during the newborn period may be widespread and fairly severe, although this does not indicate the phenotype that will develop later in life [9]. Mucous membrane erosions, onychodystrophy, post-inflammatory hyperpigmentation, acral blistering, occasionally in a circinate distribution, late-onset palmoplantar keratoderma, and acral blistering are all rather prevalent [2]. Autosomal recessive mutations in the PLEC gene cause severe types of EBS [10,11].

Due to the DST-encoded isoforms' tissue-specific expression and function, mutations in the DST gene may result in either a neuronal or a skin phenotype. Three homozygous deleterious DST mutations have been identified thus far, two of which are nonsense mutations that affect the skin isoform (DST-e, NM 001723; c.3478C>T, p.Q1124X, and c.3853A>T, p.R1249X, both in exon 23) and one of which is a one-base deletion that affects the brain isoform (DST-a, NM_001144769; c.15399delA in exon 86).

Multiple studies have connected anomalies in the DST gene and BPAG1-e protein to disorders of the nervous system in humans. Notably, there is an epidemiological link between individuals with bullous pemphigoid, a subepidermal blistering illness, and an increased incidence of multiple sclerosis, Parkinson's disease, epilepsy, dementia, and stroke [12]. Our patient did not, however, present with any neurological manifestations.

The patient did not undergo either echocardiography (ECHO) or GI endoscopy. As per the patient's history and her clinical presentation, there was no indication or symptoms suggestive of cardiac or GI involvement. The patient did not show signs of feeding difficulties, GI bleeding, or recurrent abdominal pain, which might have warranted a GI endoscopy. Similarly, there was no history of cardiac symptoms such as palpitations, shortness of breath, or chest pain to warrant an ECHO. The disease manifestation in this patient was primarily limited to the skin. Should the patient develop any relevant symptoms in the future, appropriate investigations, including ECHO or GI endoscopy, will be considered.

The prognosis varies, depending on the severity of the disease. Some patients have a relatively mild course, while others may have severe and disabling symptoms. Severe cases can lead to significant morbidity and early mortality, particularly when the condition is associated with internal complications. Novel therapies are being investigated. Gene therapy, which involves replacing the mutated gene with a healthy one, is a promising approach, though it is still in the experimental stages. Protein replacement therapy, providing the body with the DST it lacks, is another area of exploration. Since homozygous nonsense mutations in the DST gene are inherited in an autosomal recessive manner, genetic counseling should be provided to affected individuals and their families [13].

## Conclusions

The management of individuals with EB should involve a multidisciplinary team including a dermatologist, primary care provider, occupational therapist, nutritionist, and social worker. The treatment of those patients is largely supportive and includes control of any infection, wound care, nutritional support, and treatment of complications. Early dental care is crucial for reducing the development of caries and improving oral health. The patients should be encouraged to consume less cariogenic foods as well. Consultation of other medical specialties e.g., gastroenterology, nephrology, endocrinology, and plastic surgery might sometimes be needed.

## Appendices

**Table 1:** Cases of association of DST mutations with EBS reported in the literature. DST: dystonin EBS: epidermolysis bullosa simplex

Study	Nationality	Age at diagnosis	Sex	Mutation	Onset of manifestations
He et al. [14]	Turkey	19	Male	c.2618_2620delAAG (p.Glu873del) / c.2618_2620delAAG (p.Glu873del); c.3805 C > T (p.Gln1269X) / c.3805 C > T (p.Gln1269X)	Childhood
Turcan et al. [15]	Syria	39	Male	c.6559 C > T (p.Gln2187X) / c.6559 C > T (p.Gln2187X)	Childhood
Groves et al. [16]	Kuwait	38	Male	c.3478C > T (p.Gln1124X) / c.3478C>T (p.Gln1124X)	Infancy
Liu et al. [17]	Iran	34	Female	c.3853A > T (p.Arg1249X) / c.3853A > T (p.Arg1249X)	Infancy
Takeichi et al. [18]	Kuwait	Four Kuwaiti families were identified with subjects with DST-e mutations and skin fragility		c.3370 C > T (p.Gln1124X) / c.3370 C > T (p.Gln1124X)	Infancy
Cappuccio et al. [19]	Caucasian	17	Female	c.3886 A > G (p.R1296X) / c.806 C > T (p.H269R)	12 years of age
Ganani et al. [20]	Israeli of Iraqi origin	48	Female	c.3370 C > T (p.Gln1124X) / c.3370 C > T (p.Gln1124X)	10 years of age
	Israeli of Iraqi origin	49	Female	c.3370 C > T (p.Gln1124X) / c.3370 C > T (p.Gln1124X)	10 years of age
	Israeli of Indian origin	58	Female	c.7097dupA (p.Tyr2366X) / c.7429delC (p.Leu2477Serfs*13)	10 years of age
	Israeli of Indian origin	70	Male	c.7097dupA (p.Tyr2366X) / c.7429delC (p.Leu2477Serfs*13)	20 years of age
	Israeli of Indian origin	8	Male	c.7097dupA (p.Tyr2366X) / c.7097dupA (p.Tyr2366X)	3 years of age

## References

[1] Fine JD, Bruckner-Tuderman L, Eady RA (2014). Inherited epidermolysis bullosa: updated recommendations on diagnosis and classification. J Am Acad Dermatol.

[2] Fine JD (2010). Inherited epidermolysis bullosa. Orphanet J Rare Dis.

[3] Bolton L (2022). New options to manage epidermolysis bullosa. Wounds.

[4] So JY, Teng J (1993-2023). Epidermolysis bullosa simplex. GeneReviews® [Internet].

[5] Laimer M, Prodinger C, Bauer JW (2015). Hereditary epidermolysis bullosa. J Dtsch Dermatol Ges.

[6] Has C, Bauer JW, Bodemer C (2020). Consensus reclassification of inherited epidermolysis bullosa and other disorders with skin fragility. Br J Dermatol.

[7] Fine JD, Eady RA, Bauer EA (2008). The classification of inherited epidermolysis bullosa (EB): Report of the Third International Consensus Meeting on Diagnosis and Classification of EB. J Am Acad Dermatol.

[8] Pigors M, Kiritsi D, Krümpelmann S (2011). Lack of plakoglobin leads to lethal congenital epidermolysis bullosa: a novel clinico-genetic entity. Hum Mol Genet.

[9] Murrell DF, Trisnowati N, Miyakis S, Paller AS (2011). The yin and the yang of keratin amino acid substitutions and epidermolysis bullosa simplex. J Invest Dermatol.

[10] Bolling MC, Jongbloed JD, Boven LG, Diercks GF, Smith FJ, Irwin McLean WH, Jonkman MF (2014). Plectin mutations underlie epidermolysis bullosa simplex in 8% of patients. J Invest Dermatol.

[11] Bolling MC, Pas HH, de Visser M (2010). PLEC1 mutations underlie adult-onset dilated cardiomyopathy in epidermolysis bullosa simplex with muscular dystrophy. J Invest Dermatol.

[12] Stinco G, Codutti R, Scarbolo M, Valent F, Patrone P (2005). A retrospective epidemiological study on the association of bullous pemphigoid and neurological diseases. Acta Derm Venereol.

[13] Khalesi R, Harvey N, Garshasbi M, Kalamati E, Youssefian L, Vahidnezhad H, Uitto J (2022). Pathogenic DST sequence variants result in either epidermolysis bullosa simplex (EBS) or hereditary sensory and autonomic neuropathy type 6 (HSAN-VI). Exp Dermatol.

[14] He Y, Leppert J, Steinke H, Has C (2017). Homozygous nonsense mutation and additional deletion of an amino acid in BPAG1e causing mild localized epidermolysis bullosa simplex. Acta Derm Venereol.

[15] Turcan I, Pasmooij AM, Gostyński A (2017). Epidermolysis bullosa simplex caused by distal truncation of BPAG1-e: an intermediate generalized phenotype with prurigo papules. J Invest Dermatol.

[16] Groves RW, Liu L, Dopping-Hepenstal PJ (2010). A homozygous nonsense mutation within the dystonin gene coding for the coiled-coil domain of the epithelial isoform of BPAG1 underlies a new subtype of autosomal recessive epidermolysis bullosa simplex. J Invest Dermatol.

[17] Liu L, Dopping-Hepenstal PJ, Lovell PA (2012). Autosomal recessive epidermolysis bullosa simplex due to loss of BPAG1-e expression. J Invest Dermatol.

[18] Takeichi T, Nanda A, Liu L (2015). Founder mutation in dystonin-e underlying autosomal recessive epidermolysis bullosa simplex in Kuwait. Br J Dermatol.

[19] Cappuccio G, Pinelli M, Torella A (2017). Expanding the phenotype of DST-related disorder: a case report suggesting a genotype/phenotype correlation. Am J Med Genet A.

[20] Ganani D, Malovitski K, Sarig O, Gat A, Sprecher E, Samuelov L (2021). Epidermolysis bullosa simplex due to bi-allelic DST mutations: case series and review of the literature. Pediatr Dermatol.

